# Nociceptive Sensitizers Are Regulated in Damaged Joint Tissues, Including Articular Cartilage, When Osteoarthritic Mice Display Pain Behavior

**DOI:** 10.1002/art.39523

**Published:** 2016-03-28

**Authors:** Clare Driscoll, Anastasios Chanalaris, Chancie Knights, Heba Ismail, Pradeep K. Sacitharan, Clive Gentry, Stuart Bevan, Tonia L. Vincent

**Affiliations:** ^1^ Kennedy Institute of Rheumatology and University of OxfordOxfordUK; ^2^ King's College LondonLondonUK

## Abstract

**Objective:**

Pain is the most common symptom of osteoarthritis (OA), yet where it originates in the joint and how it is driven are unknown. The aim of this study was to identify pain‐sensitizing molecules that are regulated in the joint when mice subjected to surgical joint destabilization develop OA‐related pain behavior, the tissues in which these molecules are being regulated, and the factors that control their regulation.

**Methods:**

Ten‐week‐old mice underwent sham surgery, partial meniscectomy, or surgical destabilization of the medial meniscus (DMM). Pain‐related behavior as determined by a variety of methods (testing of responses to von Frey filaments, cold plate testing for cold sensitivity, analgesiometry, incapacitance testing, and forced flexion testing) was assessed weekly. Once pain‐related behavior was established, RNA was extracted from either whole joints or microdissected tissue samples (articular cartilage, meniscus, and bone). Reverse transcription–polymerase chain reaction analysis was performed to analyze the expression of 54 genes known to regulate pain sensitization. Cartilage injury assays were performed using avulsed immature hips from wild‐type or genetically modified mice or by explanting articular cartilage from porcine joints preinjected with pharmacologic inhibitors. Levels of nerve growth factor (NGF) protein were measured by enzyme‐linked immunosorbent assay.

**Results:**

Mice developed pain‐related behavior 8 weeks after undergoing partial meniscectomy or 12 weeks after undergoing DMM. NGF, bradykinin receptors B1 and B2, tachykinin, and tachykinin receptor 1 were significantly regulated in the joints of mice displaying pain‐related behavior. Little regulation of inflammatory cytokines, leukocyte activation markers, or chemokines was observed. When tissue samples from articular cartilage, meniscus, and bone were analyzed separately, NGF was consistently regulated in the articular cartilage. The other pain sensitizers were also largely regulated in the articular cartilage, although there were some differences between the 2 models. NGF and tachykinin were strongly regulated by simple mechanical injury of cartilage in vitro in a transforming growth factor β–activated kinase 1–, fibroblast growth factor 2–, and Src kinase–dependent manner.

**Conclusion:**

Damaged joint tissues produce proalgesic molecules, including NGF, in murine OA.

Pain is the most common presenting symptom of osteoarthritis (OA), but when and where pain originates in the arthritic joint is not yet clear. The disease is characterized by significant changes in several joint tissues including the following: articular cartilage, where degradation of the tissue is seen; the bone where remodeling occurs, resulting in subchondral bone sclerosis, osteophyte formation, and bony epiphyseal expansion; the synovium, which is subject to thickening and episodic inflammation; and the joint capsule and ligaments, which may become thickened and fibrotic 1. With the exception of the articular cartilage, joint tissues are highly innervated. During disease, the cartilage itself can become aberrantly innervated 2.

Joint replacement surgery is successful for alleviating pain in the majority of patients with end‐stage OA, indicating that peripheral drivers of pain are critical for symptomatic disease. Central processes, arising from either spinal or supraspinal pathways, play a large part in pain amplification in chronic disease, leading to chronic pain syndromes. Thus, clinical management can be extremely challenging. In a small minority of patients, chronic pain fails to abate despite joint replacement surgery 3.

Epidemiologic studies highlight the complexity of pain in OA. The correlation between pain and radiographic changes (osteophyte score, joint space narrowing) is modest, and it is not unusual for patients with advanced radiographic OA to have no joint symptoms. Conversely, patients may present with knee pain with little or no radiographic evidence of OA, which often leads to diagnostic uncertainty. The presence of synovitis in an OA joint is frequently associated with painful disease, although this is most frequently seen in a joint with advanced disease where pathology in other tissues is also apparent 4, 5. There is some correlation between pain and cytokine levels in the joint, but these do not appear to be associated with structural changes 6. Thus far, the conclusions reached in previous studies have pointed to the likelihood that multiple tissues may give rise to symptoms, perhaps at different stages of disease, and that central processes are key to patient‐perceived pain severity and persistence. Many of these responses are likely to be modifiable by patient‐specific factors such as genetics, epigenetics, environment, and mental state (e.g., presence of anxiety or depression).

Murine models of disease potentially offer a simplified system in which to examine complex behavioral traits, because it is possible to control for genetic heterogeneity and environment. Moreover, because the disease is induced in a single joint, there are no concerns due to involvement of multiple joints, and behavioral responses that rely on asymmetry can be measured; these are sensitive and quantitative.

We previously demonstrated that following joint destabilization induced by cutting the medial meniscotibial ligament (destabilization of the medial meniscus [DMM]), a well‐validated model of OA, mice display 2 distinct phases of pain‐related behavior based on asymmetric stance measured by incapacitance testing. The first phase occurs directly as a result of joint surgery, is associated with significant synovitis in the joint, and is present in the sham‐operated as well as the destabilized joint. The mice then do not display pain behavior for a period of several weeks until the second phase of pain‐related behavior, approximately 11 weeks postsurgery. This occurs only in mice with the destabilized joints and not in the sham‐operated control mice 7.

We also previously demonstrated pain‐related behavior following partial meniscectomy by measuring mechanical hyperalgesia, cold allodynia, mechanical allodynia, and vocalizations in response to joint compression. Compared with DMM, partial meniscectomy induces OA with a more accelerated course. Using the partial meniscectomy model, a similar, albeit more rapid, biphasic pain‐related behavior response is seen 8.

By taking a candidate gene approach, we established that joints from mice exhibiting pain‐related behavior after undergoing DMM had increased levels of nerve growth factor (NGF) messenger RNA (mRNA), and that their pain was alleviated by neutralization of NGF using the soluble TrkA receptor 9. This identifies NGF as a key mediator of pain in murine OA, which accords well with observations in trials using anti‐NGF as an analgesic strategy in OA patients 10 and with enhanced hyperalgesia in OA rats following intraarticular NGF injection 11.

NGF is an inflammatory response gene that is typically induced by inflammatory cytokines. Mechanical injury to joint tissue is also able to induce inflammatory response genes in a cytokine‐independent manner. Indeed, mechanical injury of cartilage rapidly activates several intracellular signaling pathways including the MAP kinases (MAPKs) (ERK, p38, and JNK), Src kinases 12, 13, 14, transforming growth factor β–activated kinase 1 (TAK‐1) (Ismail H: unpublished data), NF‐κB 12, 14, and the Wnt pathway 15, 16. This type of injury response is also seen upon avulsion of the femoral cap of immature murine hip joints 17, and both lead to the induction of inflammatory response genes such as chemokines, proteases, and interleukins. One mechanism by which chondrocytes sense injury is by rapid release of fibroblast growth factor 2 (FGF‐2) from the pericellular matrix, leading to the induction of several downstream targets 13, 18, 19. Release of FGF‐2 probably accounts for much of the MAPK/ERK activation upon cartilage injury but does not account for the activation of inflammatory signals (TAK‐1, JNK, p38) upon injury 14.

In the current study, we identified pain‐sensitizing molecules in addition to NGF that are regulated in the joint when mice subjected to joint destabilization surgery develop OA‐related pain behavior, the tissues in which these molecules are regulated, and the factors that control their regulation.

## MATERIALS AND METHODS

### Animals

Mice were kept in approved animal care facilities and were housed 5 per cage in standard individually ventilated cages and maintained under a 12‐hour light/dark cycle at an ambient temperature of 21°C. The mice were fed a certified mouse diet (RM3; Special Diet Services) and water ad libitum. Animal experiments were performed following local ethics and statutory approval. Male C57BL/6J mice (for DMM) and female C57BL/6J mice (for partial meniscectomy) were obtained from Charles River at either 4 weeks (for hip avulsion experiments) or 9 weeks (for surgical joint destabilization). FGF‐2–deficient, tumor necrosis factor receptor p75 (TNFR p75)–deficient, TNFR p55–deficient, myeloid differentiation factor 88 (MyD88)–deficient, and JNK‐2–deficient mice were originally obtained from The Jackson Laboratory. Littermate or appropriately matched wild‐type (WT) mice were used as controls. Trotters (feet) from male 6–9‐month‐old pigs were obtained from a local abattoir, within 12 hours of slaughter.

### Surgical joint destabilization

Surgical joint destabilization was performed either by partial meniscectomy or by DMM, as previously described 8, 20. Sham‐operated joints or the contralateral joints of mice that underwent partial meniscectomy or DMM were used as controls. Behavioral assessments were performed weekly. Pain‐related behavior was judged to be consistent when it significantly deviated from that of sham‐operated control mice. Joints were obtained either for RNA extraction or for histologic assessment. The Osteoarthritis Research Society International OA grading system 21 was used to grade chondropathy, and results were expressed as a summed score (the sum of the 3 highest joint scores for all 4 sections of the joint). In order to separate the joint tissues, the articular cartilage, menisci, and epiphyses (after cartilage removal) were removed by microdissection under an operating microscope (for a detailed description of these methodologies, see refs. 22 and 23).

### Pain‐related behavior assessments

Prior to surgery, the mice were acclimatized to the behavioral assessment. Measurements of pain‐related behavior, as described previously 7, 8, were obtained at regular intervals after DMM (by author CD) and after partial meniscectomy (by authors CK and CG), with each assessor blinded with regard to treatment. Briefly, mechanical allodynia was assessed using application of von Frey filaments to the ipsilateral hind paw. Mechanical hyperalgesia was determined using a Ugo Basile 7200 Analgesy‐Meter; for this procedure, an increasing pressure stimulus is applied to the dorsal surface of the ipsilateral hind paw until the mouse withdraws. Cold allodynia was assessed using a Ugo Basile Cold Plate (10°C). Paw withdrawal latency was recorded as the length of time (in seconds) before the mouse withdraws. These are measures of hypersensitivity/allodynia distal to the joint. Mechanical allodynia at the joint was measured by the number of vocalizations in response to 10 forced flexions of the knee joint, and by assessment with a Linton Incapacitance Tester. When more than 1 assessment was performed in the same mouse, care was taken to ensure that the order of the assessments was the same (incapacitance, von Frey filaments, paw pressure, cold plate, and then forced knee flexion) in order to minimize the risk of sensitization in subsequent tests. A maximum of 3 tests were performed in a single session, with at least 10 minutes between each test.

### Murine hip cartilage injury

Male or female mice (6–7 weeks old, WT or knockout) were culled by cervical dislocation, and the acetabulofemoral joint was exposed by blunt dissection as described previously 17. Hips were either immediately snap‐frozen (time 0) or were cultured in serum‐free medium for up to 24 hours before snap‐freezing for RNA extraction (6 hips pooled per data point).

### Porcine cartilage injury

Trotters were washed in Virkon, and the skin was removed. For injection studies, 2 ml of pan–FGF receptor (FGFR) inhibitor (SB402451; 100 n*M*), MEK inhibitor (U0126; 5 μ*M*), p38 MAPK inhibitor (SB202190; 5 μ*M*), TAK‐1 inhibitor (5*Z*‐7‐oxozeaenol; 1 μ*M*), Src inhibitor (PP2; 10 μ*M*), or vehicle was injected into the metacarpophalangeal joint as previously described 14. Articular cartilage (∼0.5 gm) was dissected into serum‐free medium containing additional inhibitor or vehicle and cultured. Explants were snap‐frozen for RNA extraction, or medium was analyzed for NGF protein expression by enzyme‐linked immunosorbent assay (ELISA), using a commercial kit according to the manufacturer's instructions (Promega).

### RNA extraction and reverse transcription–polymerase chain reaction (RT‐PCR)

RNA extraction from whole and microdissected mouse joint tissue 22, 23 from avulsed acetabulofemoral joints 17 or from porcine metacarpophalangeal cartilage 14 was performed as described previously. Only RNA with an RNA integrity value of >8 was analyzed further. Murine RNA was analyzed on 2 custom‐made microfluidic cards (Thermo Scientific) carrying hydrolysis probes, interrogating 54 known pain‐regulating genes (Table 1; see also Supplementary Table 1, available on the *Arthritis & Rheumatology* web site at http://onlinelibrary.wiley.com/doi/10.1002/art.39523/abstract). Porcine‐derived complementary DNA was interrogated by individual hydrolysis probe assays (Thermo Scientific) (see Supplementary Table 2, http://onlinelibrary.wiley.com/doi/10.1002/art.39523/abstract).

**Table 1:** Genes regulated in whole‐joint extracts 8 weeks postsurgery in sham‐operated mice and mice subjected to partial meniscectomy^a^

Gene	Sham‐operated	Partial meniscectomy	*P*
*Bdkrb1*	1.03 ± 0.28	2.57 ± 1.09	≤0.05
*Bdkrb2*	1.01 ± 0.19	3.51 ± 0.88	≤0.01
*Has1*	1.01 ± 0.17	4.34 ± 2.12	≤0.05
*Ngf*	1.02 ± 0.15	1.59 ± 0.30	≤0.05
*Npy*	1.02 ± 0.23	2.16 ± 0.65	≤0.05
*Tac1*	1.12 ± 0.64	2.33 ± 0.69	≤0.05
*Tacr1*	1.05 ± 0.36	2.69 ± 0.81	≤0.05
*Tnfa*	1.00 ± 0.06	1.51 ± 0.17	≤0.01
*Trpv4*	1.02 ± 0.25	1.39 ± 0.13	≤0.05
*Vegfa*	1 ± 0.02	1.27 ± 0.13	≤0.05
*Calca*	1.14 ± 0.76	1.34 ± 0.45	NS
*Ccl2*	1.04 ± 0.32	1.35 ± 0.74	NS
*Ccl19*	1.03 ± 0.32	1.39 ± 0.25	NS
*Ccr2*	1.02 ± 0.24	0.96 ± 0.22	NS
*Ccr7*	1.01 ± 0.18	1.13 ± 0.13	NS
*Cd14*	1.02 ± 0.26	1.22 ± 0.30	NS
*Cd68*	1.03 ± 0.28	1.14 ± 0.21	NS
*Cnr1*	1.02 ± 0.27	0.91 ± 0.22	NS
*Cnr2*	1.01 ± 0.18	1.26 ± 0.13	NS
*Gal*	1.03 ± 0.29	1.08 ± 0.30	NS
*Gdnf*	1.00 ± 0.14	1.38 ± 0.48	NS
*Il1a*	1.04 ± 0.30	0.93 ± 0.21	NS
*Il1b*	1.03 ± 0.34	2.40 ± 2.70	NS
*Il1r1*	1.01 ± 0.24	2.79 ± 2.38	NS
*Il10*	1.06 ± 0.44	1.37 ± 0.21	NS
*Il15*	1.01 ± 0.24	1.21 ± 0.14	NS
*Il2*	1.13 ± 0.74	1.25 ± 0.89	NS
*Il4*	1.03 ± 0.27	1.31 ± 0.21	NS
*Il6*	1.03 ± 0.28	1.07 ± 0.45	NS
*Il6ra*	1.01 ± 0.16	1.23 ± 0.15	NS
*Nos2*	1.04 ± 0.34	1.09 ± 0.28	NS
*Nrtn*	1.09 ± 0.57	1.69 ± 0.39	NS
*Ntf3*	1.01 ± 0.19	0.97 ± 0.15	NS
*Ntf5*	1.08 ± 0.53	0.84 ± 0.28	NS
*Penk*	1.05 ± 0.38	1.09 ± 0.15	NS
*Pspn*	1.14 ± 0.76	1.34 ± 0.79	NS
*Ptg2s2*	1.06 ± 0.38	0.88 ± 0.62	NS
*Trpa1*	1.02 ± 0.24	1.24 ± 0.23	NS
*Trpv1*	1.02 ± 0.28	1.44 ± 1.03	NS

a RNA was extracted from whole knee joints at the onset of pain‐related behavior. Reverse transcription–polymerase chain reaction was performed using custom‐made TaqMan microfluidic cards for preselected genes (see Supplementary Table 1, available on the *Arthritis & Rheumatology* website at http://onlinelibrary.wiley.com/doi/10.1002/art.39523/abstract). Values are the mean ± SD fold change (n = 3 mice subjected to sham surgery; n = 4–5 mice subjected to partial meniscectomy). Gene expression was normalized to 18S ribosomal RNA and expressed relative to values for sham‐operated mice. *P* values were determined by 2‐tailed *t*‐test. NS = not significant.

### Statistical analysis

All groups of data were assessed for approximation to the Gaussian distribution using the D'Agostino and Pearson omnibus test of normality. Distributions were considered to be Gaussian if the *P* value for the null hypothesis was greater than 0.05. When multiple comparisons between multiple end points were performed, the Bonferroni post hoc test was used to adjust for multiplicity. To derive the number of mice required and the number of samples for PCR analyses, we performed power calculations based on previously published data 8, 22. GraphPad Prism version 6 was used for statistical analysis, unless stated otherwise.

## RESULTS

### Effect of joint destabilization on pain‐related behavior

**Figure 1:**
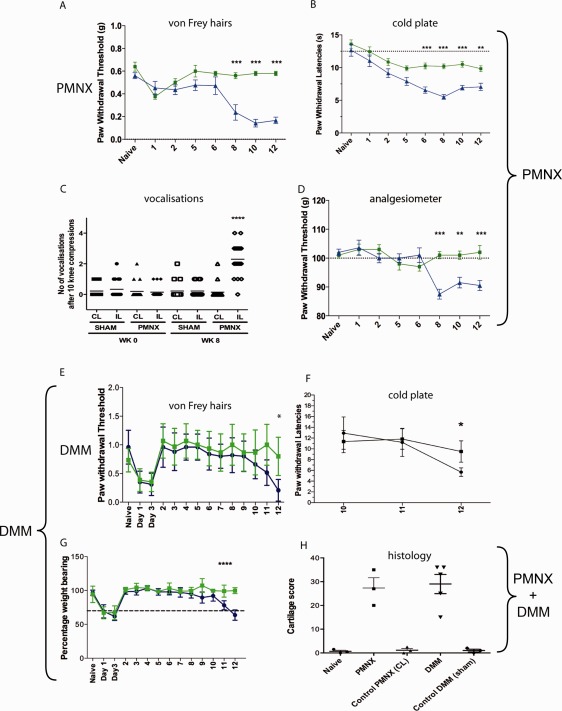
Pain behavior following joint destabilization surgery. Ten‐week‐old mice underwent surgical joint destabilization (partial meniscectomy [PMNX] [**A**–**D**] or destabilization of the medial meniscus [DMM] [**E**–**G**] [blue]) or sham surgery (green). The mice were assessed weekly until consistent pain‐related behavior was observed. **A** and **E,** Mechanical allodynia as assessed using von Frey filaments. **B** and **F,** Cold allodynia as assessed using a cold plate (10°C). **C,** Number of vocalizations in response to 10 knee compressions. Each symbol represents an individual mouse; horizontal lines show the mean**. D,** Mechanical hyperalgesia as assessed using an analgesiometer. **G,** Mechanical allodynia as assessed by incapacitance testing. **H,** Histologic scores for destabilized and sham‐operated joints at 8 weeks (PMNX) and 12 weeks (DMM) postsurgery. Values in **A,** **B,** and **D**–**H** are the mean ± SEM (n = 10 or more per group). ∗ = *P* ≤ 0.05; ∗∗ = *P* ≤ 0.01; ∗∗∗ = *P* ≤ 0.001; ∗∗∗∗ = *P* ≤ 0.0001 by two‐way analysis of variance followed by the Bonferroni post hoc test. CL = contralateral (control); IL = ipsilateral.

We first confirmed the development of pain‐related behavior following joint destabilization, using 2 different models: partial meniscectomy and DMM. Figure 1 shows that pain assessment measurements differentiated sham‐operated mice from mice that underwent partial meniscectomy or DMM‐operated mice at 8 weeks and 12 weeks postsurgery, respectively. These observations are in accordance with those findings of previous studies 7, 8. All pain assessments including mechanical allodynia, thermal hyperalgesia, and mechanical hyperalgesia showed a similar temporal trend, even though some are measuring sensitivity at a site distal to the joint (von Frey test, analgesiometry, cold plate test), and some are measuring sensitivity at the joint itself (vocalizations, Linton incapacitance test). The time of onset and the persistence of pain‐related behavior after DMM (up to 20 weeks) were confirmed by incapacitance testing in several repeat studies (data not shown). Early postoperative pain was not measured in mice that underwent partial meniscectomy, because the first behavioral assessment was performed at week 1 (after postoperative synovitis has largely resolved).

Histologic analysis of the joints was performed at the time of onset of painful behavior (week 8 for partial meniscectomy and week 12 for DMM). The chondropathy score was similar for both models and indicated significant cartilage loss, extending at least to the tidemark and often to the subchondral bone. Formal scoring of synovitis was not performed, because it was deemed to be unreliable for coronal sections; however, no increased joint inflammation was observed in mice with pain‐related behavior. Gene expression profiling of the joint was performed subsequently to investigate inflammatory gene changes within the joint.

### Regulation of pain‐sensitizing molecules in the joints of mice with pain‐related behavior

Next, we designed microfluidic cards that included hydrolysis probe assays for 54 genes known to be involved in pain sensitization. These included a number of neuropeptides and neurotrophic factors, e.g., NGF, neuropeptide Y, tachykinin (also known as substance P), their receptors, as well as several inflammatory molecules (e.g., chemokines, cytokines, and leukocyte activation markers).

Of the 54 genes examined, expression of only 39 was detected in whole‐joint extracts of mice that underwent partial meniscectomy (8 weeks postsurgery) (Table 1). Of these genes, 10 were up‐regulated in joints subjected to partial meniscectomy compared with sham‐operated joints (Table 1). These genes included *Ngf* (as expected based on the results of our previous study [9]) as well as *Tac1*, *Tacr1*, and the bradykinin receptors (*Bdkrb1* and *Bdkrb2*). *Trpv4* and *Vegfa* were significantly but weakly up‐regulated (<1.5‐fold). Interestingly, of all the inflammatory genes tested, only *Tnfa* was modestly regulated above the levels in sham‐operated mice (1.51‐fold). There was no increase in *Il1* or any chemokine or leukocyte activation marker associated with general inflammation nor any specifically implicated in neuronal sensitization (e.g., *Ccl2*) 24. The full list of 54 genes, including the 15 genes whose expression products were not detected in the joint, are available in Supplementary Table 1 (http://onlinelibrary.wiley.com/doi/10.1002/art.39523/abstract).

### Tissue localization of pain‐sensitizing molecules

We sought to determine where in the joint these pain‐sensitizing molecules were being regulated. The experiment was repeated, with the following modifications: joint tissues were microdissected (articular cartilage, epiphysis, and meniscus) and, when necessary, pooled for RNA extraction according to our previously described protocol 22, 23. The same 54 genes were examined by quantitative RT‐PCR, using microfluidic cards. Table 2 shows the genes that were differentially up‐regulated in the separated joint tissues of mice that displayed pain‐related behavior following partial meniscectomy.

**Table 2:** Regulation of pain‐sensitizing genes in microdissected tissue from mice subjected to partial meniscectomy or DMM surgery^a^

	*Bdkrb1*	*Bdkrb2*	*Tac1*	*Tacr1*	*Ngf*
Partial meniscectomy					
Cartilage	4.47 ± 2.16	2.07 ± 1.34	5.43 ± 2.68	6.89 ± 1.91	4.91 ± 2.95
*P*	≤0.05	NS	≤0.05	≤0.001	≤0.05
Meniscus	2.79 ± 0.72	0.95 ± 0.39	1.32 ± 0.50	0.99 ± 0.31	1.17 ± 0.59
*P*	≤0.01	NS	NS	NS	NS
Tibial epiphysis	2.5 ± 2.06	2.27 ± 0.30	3.95 ± 4.70	4.24 ± 3.27	1.35 ± 0.57
*P*	NS	≤0.01	NS	NS	NS
DMM					
Cartilage	142.59 ± 70.02	332.89 ± 150	0.93 ± 0.38	1.1 ± 1.07	5.94 ± 3.75
*P*	≤0.001	≤0.001	NS	NS	≤0.05
Meniscus	4.17 ± 1.14	1.82 ± 0.345	3.33 ± 0.7	4.11 ± 1.54	2.34 ± 0.69
*P*	≤0.005	≤0.01	≤0.001	≤0.001	≤0.05
Tibial epiphysis	2.94 ± 0.95	3.11 ± 2.04	13.88 ± 12.14	2.86 ± 1.49	1.38 ± 0.95
*P*	≤0.01	NS	NS	≤0.001	NS

a RNA was extracted from microdissected tissue obtained from mice that underwent partial meniscectomy (n = 3), mice that underwent surgical destabilization of the medial meniscus (DMM) (n = 6), and sham‐operated mice at 8 weeks postsurgery (partial meniscectomy group) or 20 weeks postsurgery (DMM group). Reverse transcription–polymerase chain reaction was performed using custom‐made TaqMan microfluidic cards. Gene expression was normalized to the 18S ribosomal RNA housekeeping gene and expressed relative to levels in sham‐operated mice. Values are the mean ± SD fold change. *P* values were determined by 2‐tailed *t*‐test. NS = not significant.

Of the 10 genes regulated in whole joints, 5 were regulated in microdissected tissue from the joints of mice exhibiting pain‐related behavior compared with sham‐operated controls (no pain). Furthermore, gene regulation occurred largely in the articular cartilage rather than in the bone or meniscus. These genes included *Bdkrb1, Tac1*, *Tacr1*, and *Ngf*. *Bdkrb1* was also regulated in the meniscus. *Bdkrb2* was regulated only in the tibial epiphysis. Genes that were regulated in the whole joint but not regulated in the cartilage, meniscus, or bone included *Has1*, *Npy*, *Tnfa*, *Trpv4*, and *Vegf*. It is possible that these molecules were regulated in the synovium, which is a tissue that we have not been able to examine separately in this type of analysis.

To determine the robustness of these observations, we repeated the experiment in the DMM model of OA and analyzed RNA from the microdissected tissue of sham‐operated or DMM‐operated joints at a time when the mice had pain‐related behavior that had been constant for several weeks (20 weeks postsurgery) (Table 2). Of the 5 pain sensitizers regulated in the joint tissue of mice subjected to partial meniscectomy, all were up‐regulated in tissue from DMM‐operated joints compared with sham‐operated joints. *Bdkrb1*, *Bdkrb2*, and *Ngf* were strongly and significantly up‐regulated in articular cartilage from DMM‐operated joints. Unlike what we observed in mice that underwent partial meniscectomy, all of the genes examined were regulated in the meniscus of DMM‐operated joints. Only *Tacr1* and *Bdkrb1* were regulated in the bone.

### Strong regulation of pain‐sensitizing molecules by cartilage injury in vitro

**Figure 2:**
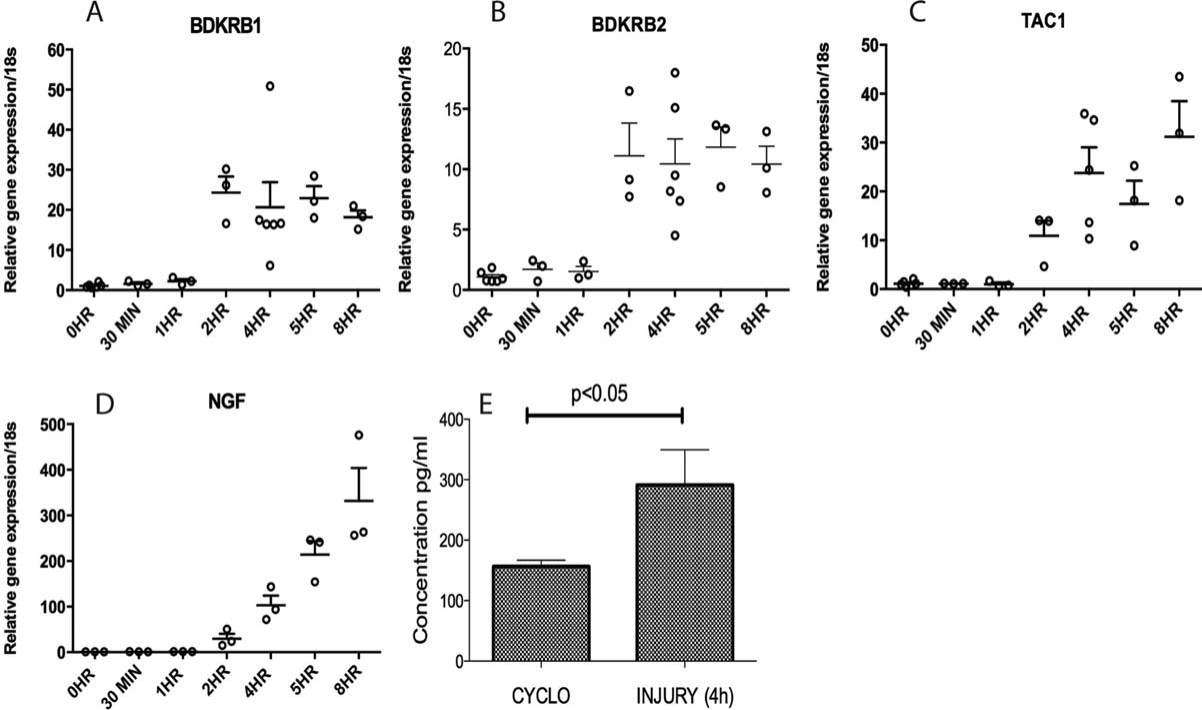
Induction of pain‐sensitizing molecules in response to cartilage injury. Murine hips (6 hips pooled for each experimental data point) or articular cartilage explants from porcine joints were either snap‐frozen or cultured for up to 8 hours in serum‐free medium or cycloheximide (Cyclo). **A–D,** Changes over time in expression of *Bdkrb1* (**A**), *Bdkrb2* (**B**), *Tac1* (**C**), and *Ngf* (**D**), as determined by reverse transcription–polymerase chain reaction. Gene expression was normalized to 18S ribosomal RNA and expressed relative to time 0. Values are the mean ± SEM fold change (n = 3–6 experimental data points). In **A–C**, values at 2 hours, 4 hours, 5 hours, and 8 hours were significant versus time 0 (*P* < 0.001 in **A** and **B**; *P* < 0.0001 in **C**) and at 4, 5, and 8 hours versus time 0 in **D** (*P* < 0.001) by one‐way analysis of variance. **E,** NGF protein secretion from injured porcine cartilage explants as determined by enzyme‐linked immunosorbent assay. Values are the mean ± SEM.

Because most of the genes were regulated in damaged articular cartilage in vivo, we sought to determine whether cartilage injury per se was sufficient to induce this regulation. We used a previously described murine cartilage injury assay in which the cartilaginous femoral head is avulsed from the femur of 5‐week‐old mice 17. Figures 2A–D show the induction of *Bdkrb1*, *Bdkrb2*, *Tac1*, and *Ngf* in response to avulsion injury. Apart from *Tacr1* (data not shown), all of the genes were robustly induced by cartilage injury, and regulation was first evident 2 hours after injury. Induction of *Ngf* was particularly strong, peaking at ∼300‐fold (compared with time 0 levels) 8 hours after injury (Figure 2D). Increased NGF protein secretion following explantation injury was detected by ELISA (Figure 2E). Cycloheximide was added to the control explants to indicate that NGF accumulation after injury was attributable to new protein synthesis. The pathways driving *Bdkrb1*, *Bdkrb2*, *Tac1*, and *Ngf* regulation upon injury were then examined further.

### Induction of *Ngf* and *Tac1* upon cartilage injury is FGF‐2–, TAK‐1–, and Src kinase–dependent

To investigate the molecular basis for regulation of pain sensitizers following cartilage injury, we used 2 models: avulsion of the hip joint from genetically modified mice 17 and explantation from an intact porcine metatarsophalangeal joint in which pharmacologic inhibitors had been injected prior to injury 14. Using a combination of these approaches, we were able to examine the involvement of *Fgf2*, *Tnfa* (in view of its regulation in the joint at the time of pain onset), and several intracellular signaling pathways that are known to be activated in response to cartilage injury. Table 3 summarizes the data for murine cartilage injury and shows that regulation of *Tac1* and *Ngf* upon injury is significantly dependent on FGF‐2. *Bdkrb1* and *Bdkrb2* induction upon injury was dependent on MyD88 (an adapter protein involved in interleukin‐1 and Toll‐like receptor signaling) rather than being FGF‐2–dependent, with MyD88 being a negative regulator of gene expression. TNFR p75 and TNFR p55 did not influence regulation of any of the genes tested upon cartilage injury.

**Table 3:** Pathways driving regulation of pain‐sensitizing genes upon cartilage injury in mice^a^

Strain	*Bdkrb1*	*Bdkrb2*	*Tac1*	*Ngf*
p75				
WT	4.94 ± 7.28	7.15 ± 10.65	4.47 ± 1.50	56.38 ± 96.24
Knockout	8.76 ± 6.89	7.17 ± 5.65	3.57 ± 1.86	69.61 ± 38.88
Knockout/WT	1.77	1.02	0.79	1.23
p55				
WT	24.43 ± 13.01	18.99 ± 9.98	28.22 ± 31.42	336.8 ± 235.2
Knockout	17.69 ± 13.67	16.36 ± 18.44	11.51 ± 9.55	223.4 ± 276.4
Knockout/WT	0.72	0.86	0.41	0.66
MyD88				
WT	10.97 ± 2.19	10.15 ± 2.27	74.61 ± 46.15	92.52 ± 10.78
Knockout	29.66 ± 9	26.44 ± 4.05	45.9 ± 39.15	120.7 ± 38.66
Knockout/WT	2.7†	2.6†	0.61	1.31
FGF‐2				
WT	6.29 ± 1.99	6.05 ± 0.56	14.11 ± 7.63	152.50 ± 46.67
Knockout	8.01 ± 1.3	6.12 ± 1.49	6.03 ± 2.38	59.65 ± 21.17
Knockout/WT	1.27	0.98	0.427†	0.39†

a Hip cartilage specimens obtained from wild‐type (WT) or genetically modified (knockout) mice (p75^−/−^, p55^−/−^, MyD88^−/−^, FGF‐2^−/−^) were avulsed into serum‐free medium for 4 hours. RNA was extracted (samples from 6 hips pooled for each experimental data point [n = 3]). Gene expression was normalized to 18S ribosomal RNA and expressed relative to levels for WT mice. *P* values were determined by one‐way analysis of variance followed by the Bonferroni post hoc test. Values are the mean ± SEM fold change at 4 hours relative to 0 hours. MyD88 = myeloid differentiation factor 88; FGF‐2 = fibroblast growth factor 2.

*P* < 0.05.

**Figure 3:**
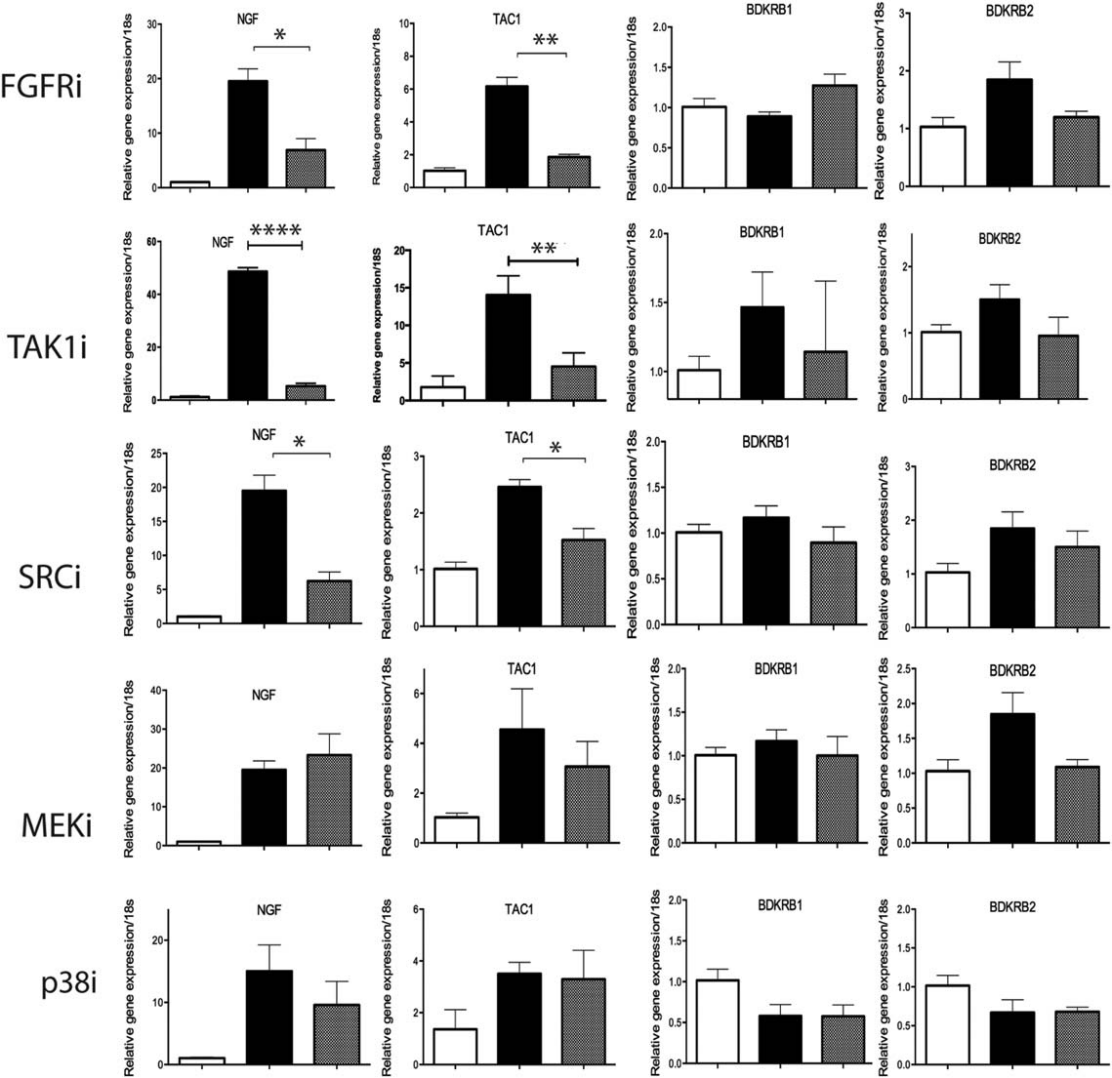
Pathways driving regulation of *Ngf*, *Tac1*, *Bdkrb1*, and *Bdkrb2* upon porcine cartilage injury. Porcine metacarpophalangeal joints were injected with inhibitors or vehicle 2 hours prior to cartilage explantation. Explants were either snap‐frozen (open bars) or cultured for 4 hours in serum‐free medium containing vehicle (solid bars) or inhibitor (shaded bars). The inhibitors included a pan–fibroblast growth factor receptor inhibitor (FGFRi) (SB402451; 100 n*M*), a transforming growth factor β–activated kinase 1 inhibitor (TAK‐1i) (5*Z*–7‐oxozeaenol; 1 μ*M*), a Src inhibitor (SRCi) (PP2; 10 μ*M*), a MEK inhibitor (MEKi) (U0126; 5 μ*M*), and a p38 MAPK inhibitor (p38i) (SB202190; 5 μ*M*). RNA was extracted, and reverse transcription–polymerase chain reaction was performed. Gene expression was normalized to 18S ribosomal RNA and expressed relative to time 0. Bars show the mean ± SEM fold change (n = 3). ∗ = *P* ≤ 0.05; ∗∗ = *P* ≤ 0.01; ∗∗∗∗ = *P* ≤ 0.0001 by one‐way analysis of variance followed by the Bonferroni post hoc test.

Figure 3 shows the results of pharmacologic inhibitor studies of porcine cartilage injury. Gene regulation upon porcine cartilage injury was generally less pronounced, and *Bdkrb1* and *Bdkrb2* were inconsistently regulated 4 hours after injury. Regulation of *Ngf* and *Tac1* was robust, and both genes demonstrated dependence on FGF‐2, TAK‐1, and Src but did not demonstrate dependence on ERK or p38 MAPK.

Because *Ngf* regulation was significantly dependent on FGF‐2, we speculated that pain‐related behavior might be delayed in FGF‐2–null mice following induction of OA. When incapacitance testing was performed in FGF‐2–null mice following DMM surgery, rather than displaying delayed onset of pain‐related behavior, these mice displayed pain‐related behavior 4 weeks earlier than WT mice (∼7 weeks postsurgery) (see Supplementary Figure 1A, available on the *Arthritis & Rheumatology* web site at http://onlinelibrary.wiley.com/doi/10.1002/art.39523/abstract). Similar chondropathy scores were observed at the time of onset of pain‐related behavior for both groups of mice (see Supplementary Figure 1B, http://onlinelibrary.wiley.com/doi/10.1002/art.39523/abstract).

## DISCUSSION

We previously demonstrated that surgical models of OA can be used successfully to investigate pain responses and mechanisms of pain in vivo 7, 8. In the current study, we present data for 2 models of OA induced by surgical joint destabilization, showing that several pain‐sensitizing molecules, including *Ngf*, *Bdkrb1*, *Bdkrb2*, *Tac1*, and *Tacr1*, are regulated within the joints of mice at the time they display pain‐related behavior. The relative importance of these molecules in driving pain‐related behavior is unclear. Previous neutralizing experiments with soluble TrkA performed by our group have highlighted a key role for NGF as an analgesic target in murine OA 9. Other investigators have shown this is also true for bradykinin receptor targeting 25. The targeting of *Tac1* has not, to our knowledge, been examined in OA models.

When gene regulation was examined at the level of the individual tissues, several of the genes, including *Ngf*, were regulated within the articular cartilage in vivo; articular cartilage is a tissue that is generally not thought to be involved directly in pain sensitization. Some pain sensitizers were also regulated in bone, although this was not the case for *Ngf* or *Tac1*. Although the same 5 genes were regulated in both models of OA, there were some differences in the tissue specificity of this regulation. Gene regulation in the meniscus was very different between the 2 models, most likely because most of the load‐bearing meniscus had been removed following partial meniscectomy. Subtle differences between the models might also be explained by the timing of tissue sampling (8 weeks for partial meniscectomy [pain onset] and 20 weeks for DMM [established pain]) and by the sex of the mice studied (female mice for partial meniscectomy and male mice for DMM).

Even though the induction of nociceptive sensitizers is strongly linked to inflammation 26, there was little inflammatory response in the joint at the time the mice demonstrated pain‐related behavior; with modest regulation of *Tnfa* (1.5‐fold that observed in sham‐operated joints) but no regulation of *Il1*, *Il6*, leukocyte activation markers, or chemokines such as *Ccl2*. These inflammatory response genes are strongly regulated immediately following destabilization surgery when synovitis is also present, but this regulation does not persist beyond 2 weeks 22. We were also unable to detect inflammatory gene regulation in the dorsal root ganglia at the time of pain‐related behavior (data not shown). Along with our previous data showing that anti‐TNF treatment does not alter pain‐related behavior in DMM‐operated mice 9, these results suggest that synovitis is not the principal driver of pain‐related behavior in these animals. Nonetheless, we are unable to exclude a minor role for synovitis at the onset of pain‐related behavior in murine OA.

The results presented here reveal that mechanical injury is sufficient to regulate pain‐sensitizing molecules in cartilage in vitro and raise the possibility that mechanical injury may be a key driver of pain sensitization in OA. This is consistent with the observation that the chondropathy score at the onset of pain‐related behavior is comparable irrespective of the OA model. Using a number of well‐validated cartilage injury models in combination with genetically modified tissues and pharmacologic inhibitors, we unraveled some of the cellular pathways responsible for induction of pain sensitizers upon injury. The regulation of both *Ngf* and *Tac1* was in part dependent on FGF‐2, TAK‐1, and Src kinase. Interestingly, when we studied pain‐related behavior in FGF‐2–deficient mice with surgically induced OA, the lack of FGF‐2 did not delay pain‐related behavior. In fact, earlier onset of pain‐related behavior was observed, which most likely was related to the more rapid disease course in these mice 20. In this case, the degree of cartilage damage in knockout mice at the onset of pain‐related behavior was similar to that observed in WT mice, which demonstrates once again that pain‐related behavior is related to the chondropathy score. Regulation of *Ngf* and *Tac1* was strongly dependent on TAK‐1, suggesting that the TAK‐1 pathway may prove to be more clinically important. Regulation of *Bdkrb1* and *Bdkrb2* upon cartilage injury appeared to be quite distinct, being MyD88 dependent (negatively regulated) but not FGF‐2 dependent. The interpretation of these results is somewhat limited by the fact that the regulation of these genes in porcine cartilage was weak, thus making the results of inhibitor studies difficult to interpret.

NGF and NGF receptor regulation in chondrocytes has been documented in other studies 27, 28 and has been described in human OA cartilage, at both the protein and mRNA levels 2, 29, 30, 31. This is also the case for bradykinin receptors and the tachykinin pathway 25, 32. The expression of high‐affinity and low‐affinity receptors for neurotrophic factors such as NGF in chondrocytes raises the possibility that regulation of these molecules upon injury may have direct effects on cartilage in addition to sensitization of nociceptive fibers. Diverse functional roles for NGF have been described in chondrocytes and other tissues 33, 34, 35, 36, 37, 38, 39, and a disease‐modifying effect of NGF in OA was suggested after accelerated disease developed in a small subset of patients treated with a neutralizing antibody to NGF 10, 40, 41, 42.

An unexplained conundrum arising from this study is why painful behavior becomes evident only after substantial cartilage damage has occurred. If pain‐sensitizing molecules are induced and secreted in response to injury, why does pain not occur at the time of earliest cartilage damage, i.e., 2–4 weeks postsurgery? One possibility is that there is enhanced sensitivity to injury in the deeper levels of the cartilage. The change in pain‐related behavior at this stage may also be related to shorter distances that nociceptive sensitizers need to travel to reach innervated tissue such as the subchondral bone. Although not yet demonstrated, pathologic innervation of the cartilage may be developing in the mouse at this stage, as it does late in the course of human disease, and this could be a prerequisite for pain development 2.

## AUTHOR CONTRIBUTIONS

All authors were involved in drafting the article or revising it critically for important intellectual content, and all authors approved the final version to be published. Dr. Vincent had full access to all of the data in the study and takes responsibility for the integrity of the data and the accuracy of the data analysis.

### Study conception and design

Driscoll, Chanalaris, Gentry, Bevan, Vincent.

### Acquisition of data

Driscoll, Chanalaris, Knights, Ismail, Sacitharan, Gentry, Vincent.

### Analysis and interpretation of data

Driscoll, Chanalaris, Knights, Gentry, Bevan, Vincent.

## Supporting information


**Supplementary Figure 1** Incapacitance testing and disease scores in wild type and FGF2^‐/‐^ mice post DMM. (A) 10 week old wild type or FGF2^‐/‐^ mice underwent DMM or sham surgery (WT only). Incapacitance testing was checked 4 times in the first week then weekly thereafter. Percentage weight bearing indicates the proportion of weight borne through the operated compared with non‐operated joint. Data are expressed as mean ± SD. N=10 (B) Summed cartilage scores in WT and FGF2^‐/‐^ animals at the time at which animals first developed pain‐related behavior following DMM (WT 11 weeks, FGF2^‐/‐^ 7 weeks). N=3‐5 animals per group.

**Supplementary Table 1** List of Taqman® hydrolysis probe‐primer assays used for qPCR in murine tissues. Genes were selected because of their actions as inflammatory mediators and modulators of pain (reviewed in (1)).
**Supplementary table 2**. List of SYBR‐Green® primers used for qPCR in porcine tissues.
