# Supplementary figures and images for: Nociceptive Sensitizers Are Regulated in Damaged Joint Tissues, Including Articular Cartilage, When Osteoarthritic Mice Display Pain Behavior
Source: Arthritis Rheumatol. 2016 Mar 28;68(4):857–67. doi: 10.1002/art.39523 (PMC4979655; doi:10.1002/art.39523)

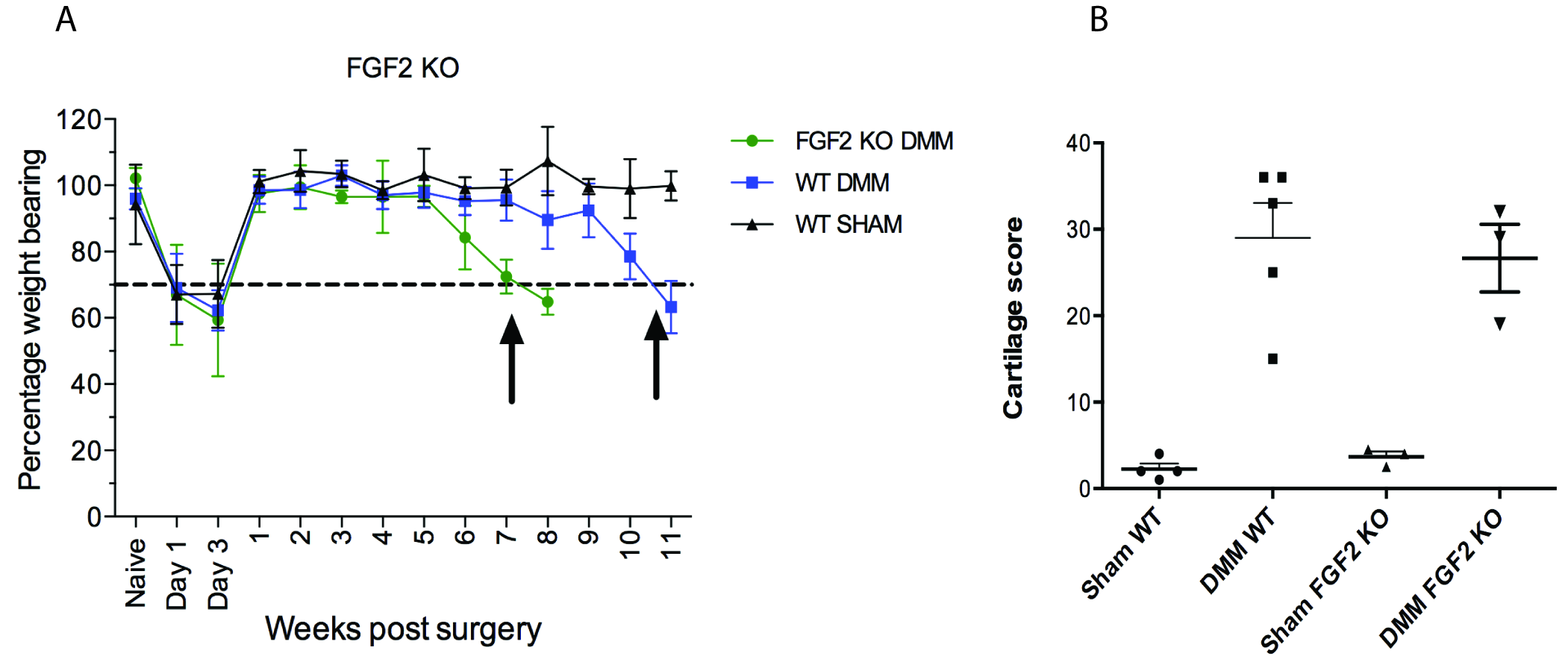

Supplement: Supplementary file 1 — Supplementary Figure 1 Incapacitance testing and disease scores in wild type and FGF2‐/‐ mice post DMM. (A) 10 week old wild type or FGF2‐/‐ mice underwent DMM or sham surgery (WT only). Incapacitance testing was checked 4 times in the first week then weekly thereafter. Percentage weight bearing indicates the proportion of weight borne through the operated compared with non‐operated joint. Data are expressed as mean ± SD. N=10 (B) Summed cartilage scores in WT and FGF2‐/‐ animals at the time at which animals first developed pain‐related behavior following DMM (WT 11 weeks, FGF2‐/‐ 7 weeks). N=3‐5 animals per group. [file ART-68-857-s001.tif]
