# Supplementary material for: Nociceptive Sensitizers Are Regulated in Damaged Joint Tissues, Including Articular Cartilage, When Osteoarthritic Mice Display Pain Behavior
Source: Arthritis Rheumatol. 2016 Mar 28;68(4):857–67. doi: 10.1002/art.39523 (PMC4979655; doi:10.1002/art.39523)
Supplement: Supplementary file 2 — Supplementary Table 1 List of Taqman® hydrolysis probe‐primer assays used for qPCR in murine tissues. Genes were selected because of their actions as inflammatory mediators and modulators of pain (reviewed in (1)). Supplementary table 2. List of SYBR‐Green® primers used for qPCR in porcine tissues. [file ART-68-857-s002.docx]

**Supplementary Table 1** List of Taqman® hydrolysis probe-primer assays used for qPCR in murine tissues. Genes were selected because of their actions as inflammatory mediators and modulators of pain (reviewed in ([1](#_ENREF_1))).

| **Symbol** | **Name** | **MGI Gene/Marker ID** | **Assay number** |
| --- | --- | --- | --- |
| Artn* | artemin | MGI:1333791 | Mm03024167_s1 |
| Bdkrb1 | bradykinin receptor, beta 1 | MGI:88144 | Mm00432059_s1 |
| Bdkrb2 | bradykinin receptor, beta 2 | MGI:102845 | Mm00437788_s1 |
| Bdnf* | brain derived neurotrophic factor | MGI:88145 | Mm01334047_m1 |
| Calca | calcitonin/calcitonin-related polypeptide, alpha | MGI:2151253 | Mm00801463_g1 |
| Ccl19 | chemokine (C-C motif) ligand 19 | MGI:1346316 | Mm00839967_g1 |
| Ccl2 | chemokine (C-C motif) ligand 2 | MGI:98259 | Mm00441242_m1 |
| Ccr2 | chemokine (C-C motif) receptor 2 | MGI:106185 | Mm01216173_m1 |
| Ccr7 | chemokine (C-C motif) receptor 7 | MGI:103011 | Mm01301785_m1 |
| Cd14 | CD14 antigen | MGI:88318 | Mm00438094_g1 |
| Cd68 | CD68 antigen | MGI:88342 | Mm00839636_g1 |
| Cnr1 | cannabinoid receptor 1 (brain) | MGI:104615 | Mm00432621_s1 |
| Cnr2 | cannabinoid receptor 2 (macrophage) | MGI:104650 | Mm00438286_m1 |
| Crh* | corticotropin releasing hormone | MGI:88496 | Mm01293920_s1 |
| Gal | galanin | MGI:95637 | Mm00439056_m1 |
| Gdnf | glial cell line derived neurotrophic factor | MGI:107430 | Mm00599849_m1 |
| Has1 | Hyaluronan synthase 1 | MGI:106590 | Mm00468496_m1 |
| Il10 | interleukin 10 | MGI:96537 | Mm00439616_m1 |
| Il13* | interleukin 13 | MGI:96541 | Mm99999190_m1 |
| Il15 | interleukin 15 | MGI:103014 | Mm00434210_m1 |
| Il17a* | interleukin 17A | MGI:107364 | Mm00439619_m1 |
| Il1a | interleukin 1 alpha | MGI:96542 | Mm00439620_m1 |
| Il1b | interleukin 1 beta | MGI:96543 | Mm01336189_m1 |
| Il1r1 | interleukin 1 receptor, type I | MGI:96545 | Mm00434237_m1 |
| Il21* | interleukin 21 | MGI:1890474 | Mm00517640_m1 |
| Il24* | interleukin 24 | MGI:2135548 | Mm00474102_m1 |
| Il2 | interleukin 2 | MGI:96548 | Mm00434256_m1 |
| Il4 | interleukin 4 | MGI:96556 | Mm00445260_m1 |
| Il6 | interleukin 6 | MGI:96559 | Mm99999064_m1 |
| Il6ra | interleukin 6 receptor, alpha | MGI:105304 | Mm00439653_m1 |
| Ngf | nerve growth factor | MGI:97321 | Mm00443039_m1 |
| Nos2 | nitric oxide synthase 2, inducible | MGI:97361 | Mm01309898_m1 |
| Npy | neuropeptide Y | MGI:97374 | Mm00445771_m1 |
| Nrtn | neurturin | MGI:108417 | Mm00435387_m1 |
| Ntf3 | neurotrophin 3 | MGI:97380 | Mm00435413_s1 |
| Ntf5 | neurotrophin 5 | MGI:97381 | Mm01701591_m1 |
| Ntrk1* | neurotrophic tyrosine kinase, receptor, type 1 | MGI:97383 | Mm01219407_m1 |
| Pdyn* | prodynorphin | MGI:97535 | Mm00457573_m1 |
| Penk | preproenkephalin | MGI:104629 | Mm01212875_m1 |
| Pomc* | pro-opiomelanocortin-alpha | MGI:97742 | Mm00435874_m1 |
| Pspn | persephin | MGI:1201684 | Mm03024006_m1 |
| Ptgs2 | prostaglandin-endoperoxide synthase 2 | MGI:97798 | Mm01307329_m1 |
| Sst* | somatostatin | MGI:98326 | Mm00436671_m1 |
| Tac1 | tachykinin 1 | MGI:98474 | Mm01166996_m1 |
| Tacr1 | tachykinin receptor 1 | MGI:98475 | Mm00436892_m1 |
| Tnf | tumor necrosis factor | MGI:104798 | Mm00443258_m1 |
| Trpa1 | transient receptor potential cation channel, subfamily A, member 1 | MGI:3522699 | Mm00625268_m1 |
| Trpm8* | transient receptor potential cation channel, subfamily M, member 8 | MGI:2181435 | Mm00454566_m1 |
| Trpv1 | transient receptor potential cation channel, subfamily V, member 1 | MGI:1341787 | Mm01246282_m1 |
| Trpv3* | transient receptor potential cation channel, subfamily V, member 3 | MGI:2181407 | Mm00454996_m1 |
| Trpv4 | transient receptor potential cation channel, subfamily V, member 4 | MGI:1926945 | Mm00499025_m1 |
| Ucn* | urocortin | MGI:1276123 | Mm00445261_m1 |
| Vegfa | vascular endothelial growth factor A | MGI:103178 | Mm01281449_m1 |
| Vip* | vasoactive intestinal polypeptide | MGI:98933 | Mm00660234_m1 |

*=Not Detected in the mouse knee (8 weeks post surgery)

1. Dawes JM AD, Bennett DLH, Bevan S, McMahon SB. Inflammatory Mediators and Modulators of Pain. In: McMahon SB KM, Tracey I, Turk DC, editor. Melzack & Wall's Textbook of Pain 6th edition. 6th Edition ed. Philadelphia USA: Elsevier; 2013. p. 48-67.

| Target | Accession number | Orientation | Primer Sequence 5’-3’ |
| --- | --- | --- | --- |
| *18s* | NM213940.1 | sense | TGTGGTGTTGAGGAAAGCAG |
|  |  | antisense | GGAATCTTGTATTGGCGAGG |
| *Ngf* | XM003481483.2 | sense | CACTGGAACTCGTATTGTACCACAA |
|  |  | antisense | GCCTGCTTGCCGTCCAT |
| *Bdkrb1* | NM001113064.1 | sense | TCTGCCGCGTGGTCAAC |
|  |  | antisense | ACCACCAGGAAGATGCTGATG |
| *Bdkrb2* | NM214146.1 | sense | CGCCTCTTTTCCGCTTTCT |
|  |  | antisense | CGGGCTCCGGGACTTG |
| *Tac1* | XM003357447.3 | sense | GATGCTGGACATGGCCAGAT |
|  |  | antisense | TCATAATTCTGCATTGCACTCCTT |

**Supplementary table 2**. List of SYBR-Green® primers used for qPCR in porcine tissues.
